# Methyl 3-[(1-benzyl-4-phenyl-1*H*-1,2,3-triazol-5-yl)formamido]­propano­ate: crystal structure, Hirshfeld surface analysis and computational chemistry

**DOI:** 10.1107/S2056989020007380

**Published:** 2020-06-09

**Authors:** Ignez Caracelli, Julio Zukerman-Schpector, Huey Chong Kwong, Edward R. T. Tiekink

**Affiliations:** a Departmento de Física, Universidade Federal de São Carlos, 13565-905 São Carlos, SP, Brazil; b Laboratório de Cristalografia, Esterodinâmica e Modelagem Molecular, Departamento de Química, Universidade Federal de São Carlos, 13565-905 São Carlos, SP, Brazil; c Research Centre for Crystalline Materials, School of Science and Technology, Sunway University, 47500 Bandar Sunway, Selangor Darul Ehsan, Malaysia

**Keywords:** crystal structure, 1,2,3-triazole, Hirshfeld surface analysis, computational chemistry

## Abstract

The title compound, C_20_H_20_N_4_O_3_, is constructed about a tri-substituted 1,2,3-triazole ring, with the substituent at one C atom flanked by the C and N atoms being a substituted amide group, and with the adjacent C and N atoms bearing phenyl and benzyl groups, respectively; the dihedral angle between the pendant phenyl rings is 81.17 (12)°, indicative of an almost orthogonal disposition. In the crystal, pairwise amide-N—H⋯O(carbon­yl) hydrogen bonds lead to a centrosymmetric dimer incorporating methyl­ene-C—H⋯π(benzene) inter­actions. The dimers are linked into a supra­molecular layer in the *ab* plane *via* methyl­ene-C—H⋯N(azo) and benzene-C—H⋯O(amide) inter­actions; the layers stack along the *c*-axis direction without directional inter­actions between them. The above-mentioned inter­molecular contacts are apparent in the analysis of the calculated Hirshfeld surface, which also provides evidence for short inter-layer H⋯C contacts with a significant dispersion energy contribution.

## Abstract 2

The title compound is constructed about a tri-substituted 1,2,3-triazole ring, with the substituent at the C atom flanked by the C and N atoms being a substituted amide group, and with the adjacent C and N atoms bearing phenyl and benzyl groups, respectively. In the crystal, pairwise amide-N—H⋯O(carbon­yl) hydrogen bonds give rise to a centrosymmetric dimer.

## Chemical context   

The title 1,2,3-triazole-5-carboxamide derivative, (I), was recently prepared and characterized from a palladium-catal­ysed amino­carbonyl­ation reaction with the use of dimethyl carbonate as a sustainable solvent (de Albuquerque *et al.*, 2019 ▸). The motivation for preparing such mol­ecules rests with the known pharmacological activity of these and related 1,2,3-triazole derivatives (Bonandi *et al.*, 2017 ▸). Unambiguous structure determination of (I) is reported herein, *via* X-ray crystallography, as is a detailed analysis of the supra­molecular association by Hirshfeld surface analysis and computational chemistry.
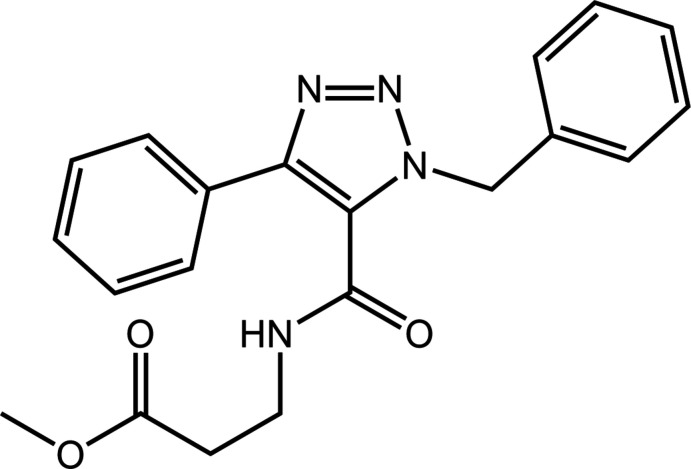


## Structural commentary   

**Figure 1:**
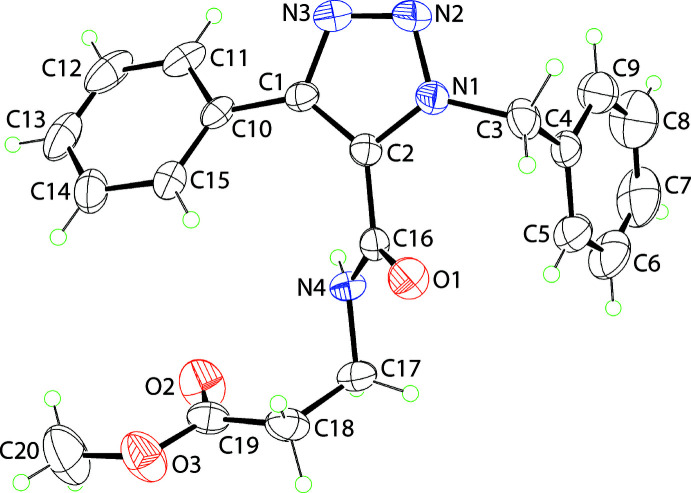
The mol­ecular structure of (I), showing the atom-labelling scheme and displacement ellipsoids at the 35% probability level.

The mol­ecular structure of (I), Fig. 1 ▸, features a tri-substituted 1,2,3-triazole ring. The five-membered ring is strictly planar with the r.m.s. deviation of the fitted atoms being 0.0021 Å. Within the ring, the lengthening of the formal azo-N2—N3 [1.306 (4) Å] and C1—C2 [1.388 (4) Å] double bonds coupled with the shortening of the N1—N2 [1.341 (4) Å], C1—N3 [1.368 (4) Å] and C2—N1 [1.347 (4) Å] bonds from their standard double/single bond values, are indicative of significant delocalization of π-electron density over the ring atoms. While the N1-bound C3-atom lies 0.131 (6) Å out of the plane of the ring, the C1- and C2-bound C10 [0.012 (6) Å] and C16 [0.008 (6) Å] atoms are effectively co-planar with the ring. The terminal residues are twisted out of the plane of the central ring as seen in the (C1,C2,N1–N3)/(C4–C9) [74.46 (13)°], (C1,C2,N1–N3)/(C10–C15) [28.10 (17)°] and (C1,C2,N1–N3)/(C16,N4,O1) [47.1 (2)°] dihedral angles. The dihedral angle between the terminal phenyl rings is 81.17 (12)° indicating a close to orthogonal disposition. There is a twist in the amide residue as seen in the value of the N4—C17—C18—C19 torsion angle of 73.6 (4)°, indicating a (+)*syn*-clinal relationship. This results in a dihedral angle close to orthogonal for the amide (C16,N4,O1) and carboxyl­ate (C19,O2,O3) residues, *i.e*. 73.6 (4)°.

## Supra­molecular features   

**Figure 2:**
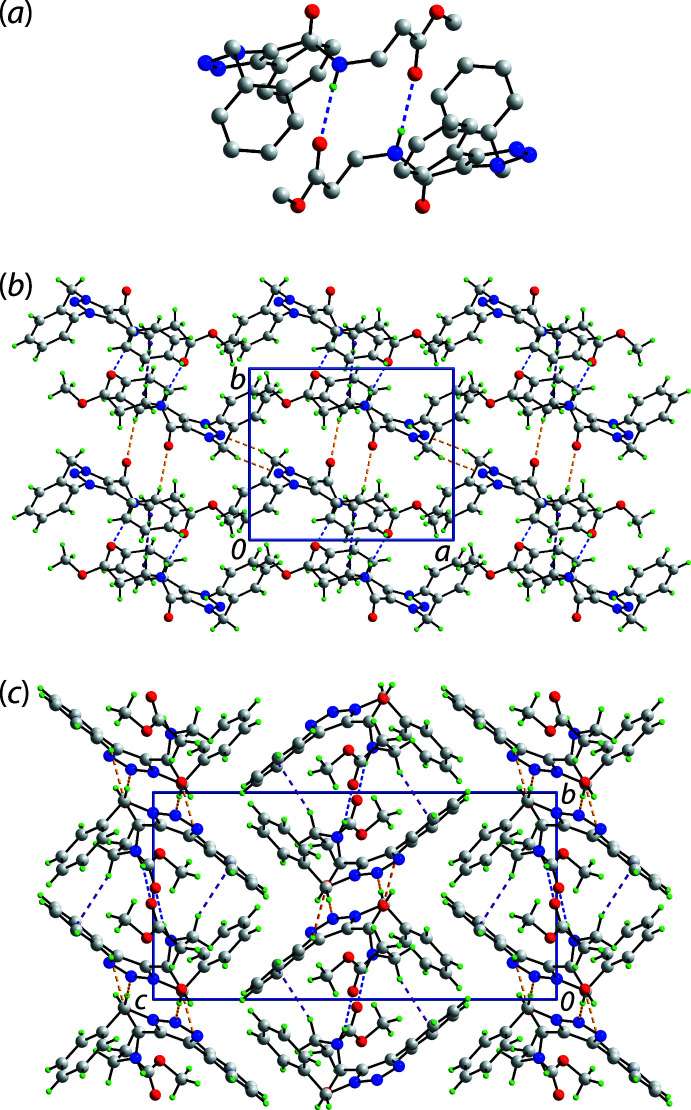
Mol­ecular packing in (I): (*a*) supra­molecular dimer sustained by amide-N4—H⋯O2(carbon­yl) hydrogen bonds (H atoms omitted for clarity), (*b*) layer where the dimers of (*a*) are connected by methyl­ene-C3—H⋯N(azo) and benzene-C15—H⋯O1(amide) inter­actions [the methyl­ene-C17—H⋯π(benzene) inter­actions occur within the dimers] and (*c*) a view of the unit-cell contents shown in projection down the *a* axis. The N—H⋯O, C—H⋯O and C—H⋯π inter­actions are shown as blue, orange and purple dashed lines, respectively.

The mol­ecular packing in (I) features several identifiable points of contact, Table 1 ▸. The most evident of these are amide-N4—H⋯O2(carbon­yl) hydrogen bonds occurring between centrosymmetrically related mol­ecules to give the dimer shown in Fig. 2 ▸(*a*). The mol­ecules in the dimer are linked *via* a 12-membered {⋯OC_3_NH}_2_ synthon and additional stability to the assembly is provided by methyl­ene-C17—H⋯π(benzene) inter­actions. The dimeric aggregates are connected into a supra­molecular layer propagating in the *ab* plane *via* methyl­ene-C3—H⋯N2(azo) and benzene-C15—H⋯O1(amide) inter­actions, Fig. 2 ▸(*b*). The layers stack in an …*ABAB*… pattern along the *c* axis and inter-digitate to potentially form π–π inter­actions. However, these are not apparent, Fig. 2 ▸(*c*). A more detailed analysis of the inter­actions occurring in the inter-layer region is provided by an analysis of the calculated Hirshfeld surfaces.

## Hirshfeld surface analysis   

**Figure 3:**
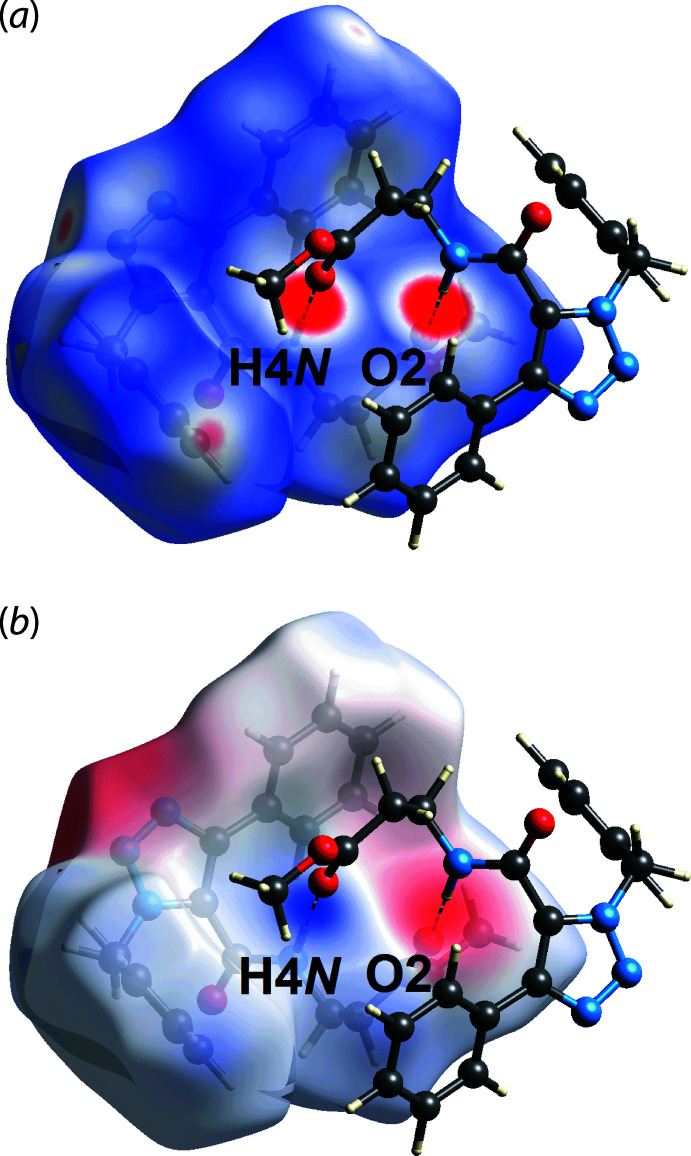
Views of the Hirshfeld surface for (I) mapped over (*a*) *d*_norm_ in the range −0.249 to +1.397 arbitrary units and (*b*) the electrostatic potential map in the range −0.097 to 0.134 atomic units, highlighting N—H⋯O hydrogen bonding.

In order to probe the inter­action between mol­ecules of (I) in the crystal, Hirshfeld surfaces mapped with the normalized contact distance *d*_norm_ (McKinnon *et al.*, 2004 ▸), electrostatic potential (Spackman *et al.*, 2008 ▸) and two-dimensional fingerprint plots were calculated using *Crystal Explorer 17* (Turner *et al.*, 2017 ▸) by established procedures (Tan *et al.*, 2019 ▸). The electrostatic potentials were calculated using the wavefunction at the HF/STO-3 G level of theory. The bright-red spots on the Hirshfeld surface mapped over *d*_norm_ in Fig. 3 ▸(*a*), *i.e*. near the amide-H4*N* and carbonyl-O2 atoms, correspond to the amide-N—H4*N*⋯O2(carbon­yl) hydrogen bond (Table 1 ▸). This hydrogen bond is also reflected in Hirshfeld surface mapped over the electrostatic potential Fig. 3 ▸(*b*), where the blue (positive electrostatic potential) and red (negative electrostatic potential) regions are apparent around the amide-H4*N* and carbonyl-O2 atoms, respectively.

**Figure 4:**
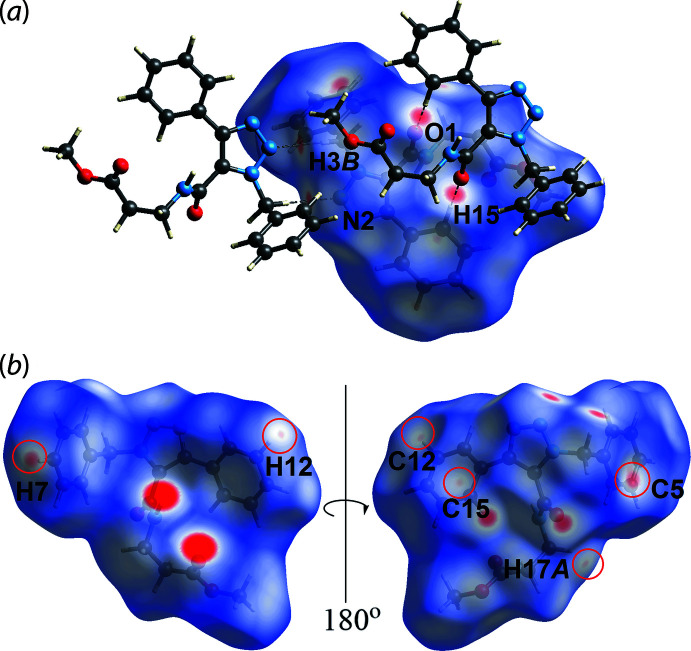
Views of the Hirshfeld surface mapped over *d*_norm_ for (I) in the range −0.249 to +1.397 arbitrary units, highlighting (*a*) weak C—H⋯N and C—H⋯O inter­actions and (*b*) short H⋯C contacts, highlighted within red circles.

**Figure 5:**
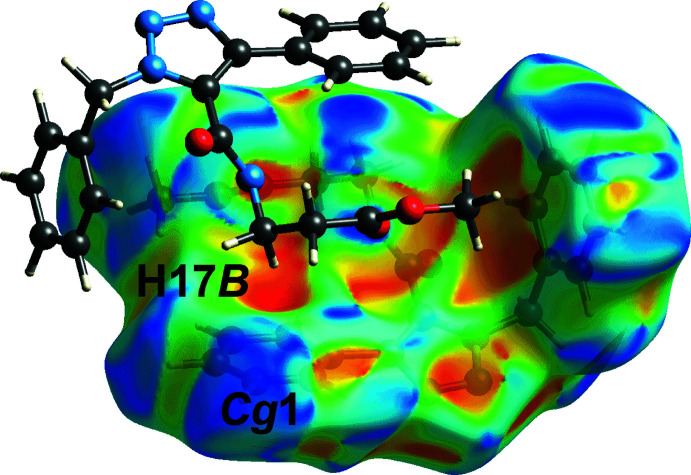
A view of the Hirshfeld surface for (I) mapped with the shape-index property, highlighting the inter­molecular C—H⋯π inter­action.

The methyl­ene-C3—H⋯N2(azo) and benzene-C15—H15⋯O1(amide) inter­actions are observed as faint-red spots on the *d*_norm_-mapped Hirshfeld surface in Fig. 4 ▸(*a*), with a distance of ∼0.3 Å shorter than the sum of their van der Waals radii, Table 2 ▸. The other faint red spots near the benzyl (C5, C12, C15, H7 and H12) and methyl­ene (H17*A*) atoms in Fig. 4 ▸(*b*) correspond to the inter-layer H7⋯C5, H17*A*⋯C12 and H12⋯C15 short contacts listed in Table 2 ▸. Even though the C—H⋯π inter­action, Table 1 ▸, was not manifested on the *d*_norm_-mapped Hirshfeld surface, this inter­action shows up as a distinctive orange ‘pothole’ on the shape-index-mapped Hirshfeld surface, Fig. 5 ▸.

**Figure 6:**
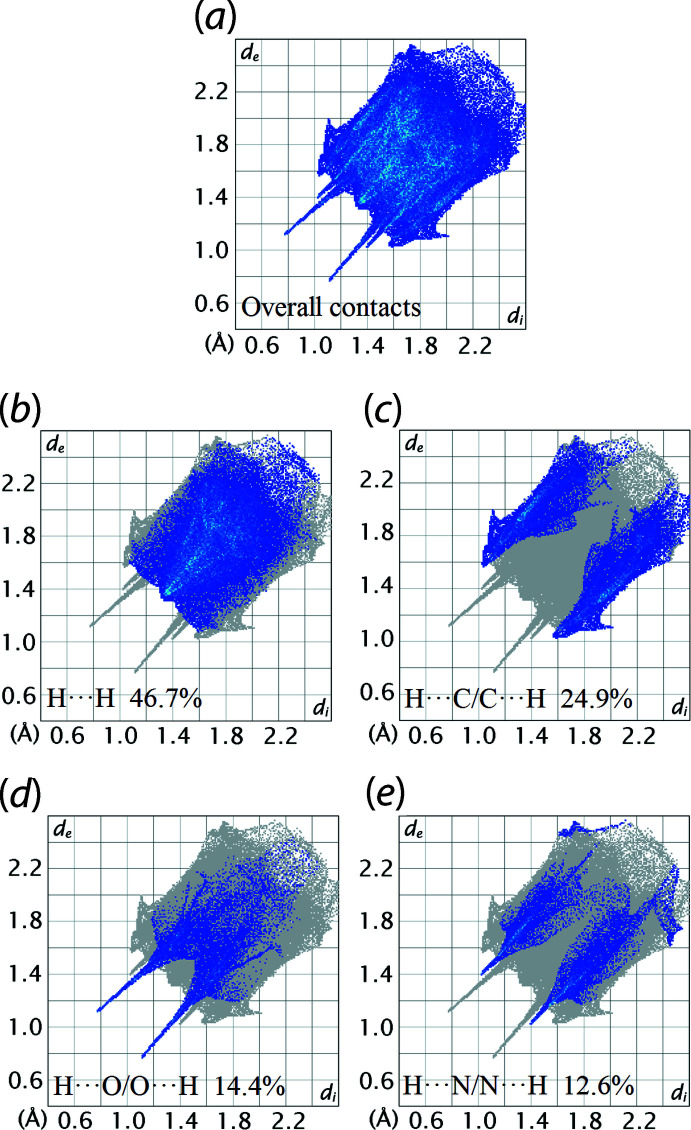
(*a*) The full two-dimensional fingerprint plot for (II) and (*b*)–(*e*) those delineated into H⋯H, H⋯C/C⋯H, H⋯O/O⋯H and H⋯N/N⋯H contacts, respectively.

The overall two-dimensional fingerprint plot for the Hirshfeld surface of (I) is shown with characteristic pseudo-symmetric wings in the upper left and lower right sides of the *d*_e_ and *d*_i_ diagonal axes, respectively, in Fig. 6 ▸(*a*). The delin­eated H⋯H, H⋯C/C⋯H, H⋯O/O⋯H and H⋯N/N⋯H contacts from the overall two-dimensional fingerprint plot are illustrated in Fig. 6 ▸(*b*)–(*e*), respectively. The percentage contributions from different inter­atomic contacts to the Hirshfeld surface of (I) are summarized in Table 3 ▸. The greatest contribution to the overall Hirshfeld surface are due to H⋯H contacts, which contribute 46.7%. However, the H⋯H contacts appear as a square-like distribution with a small beak at *d*_e_ = *d*_i_ ∼2.6 Å in Fig. 6 ▸(*b*), corresponding to H8⋯H11 ≃2.67 Å (symmetry operation: −*x*, −*y*, −*z* + 1) indicating that all H⋯H contacts have long-range characteristics. The H⋯C/C⋯H contacts on the Hirshfeld surface, which contribute 24.9% to the overall surface, Fig. 6 ▸(*c*), reflect the C—H⋯π inter­action and C⋯H short contacts as discussed above. Consistent with the C—H⋯O and C—H⋯N inter­actions occurring in the crystal, H⋯O/O⋯H and H⋯N/N⋯H contacts contribute 14.4 and 12.6%, respectively, to the overall Hirshfeld surface. These appear as two sharp symmetric spikes in the fingerprint plots at *d*_e_ + *d*_i_ ≃ 1.9 and 2.4 Å in Fig. 6 ▸(*d*) and (*e*), respectively. The contribution from the other inter­atomic contacts summarized in Table 2 ▸ has a negligible influence on the calculated Hirshfeld surface of (I).

## Energy frameworks   

**Figure 7:**
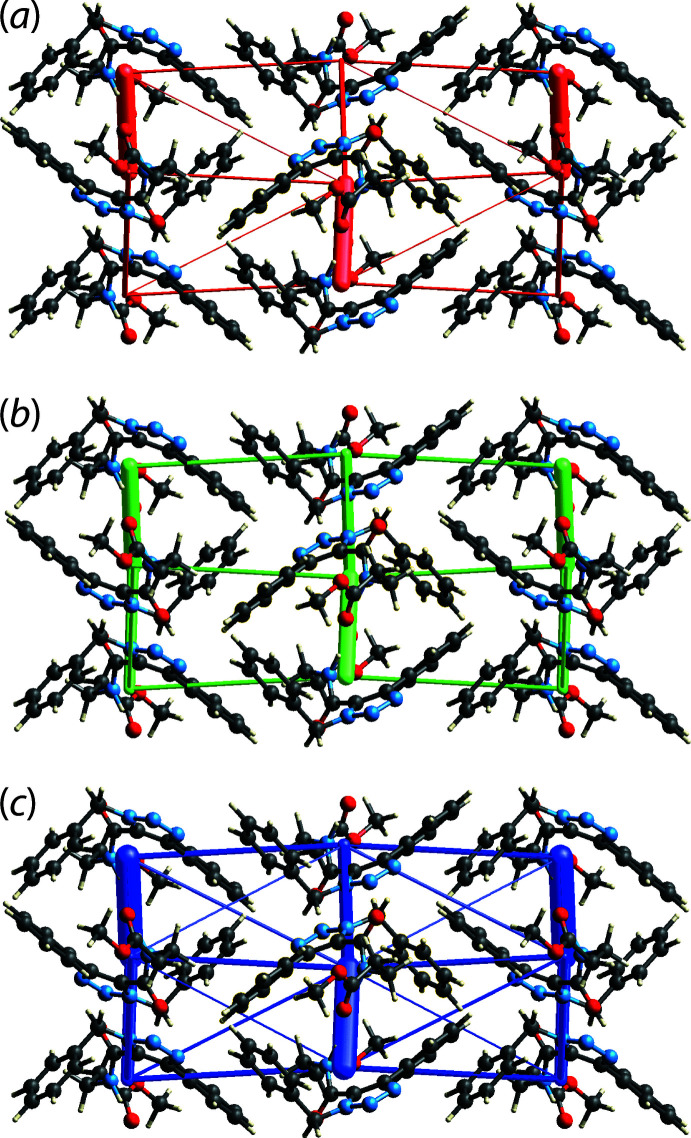
Perspective views of the energy frameworks calculated for (I) showing (*a*) electrostatic potential force, (*b*) dispersion force and (*c*) total energy, each plotted down the *a* axis. The radii of the cylinders are proportional to the relative magnitudes of the corresponding energies and were adjusted to the same scale factor of 50 with a cut-off value of 5 kJ mol^−1^ within 1 × 1 × 1 unit cells.

The pairwise inter­action energies between the mol­ecules in the crystal of (I) were calculated using the 6-31G(d,p) basis set at the B3LYP level of theory. The total energy comprises four terms, *i.e*. the electrostatic (*E*_ele_), polarization (*E*_pol_), dispersion (*E*_dis_) and exchange-repulsion (*E*_rep_) energy terms and were calculated with *Crystal Explorer 17* (Turner *et al.*, 2017 ▸). The benchmarked energies were scaled according to Mackenzie *et al.* (2017 ▸) while *E*_ele_, *E*_pol_, *E*_dis_, and *E*_rep_ were scaled as 1.057, 0.740, 0.871 and 0.618, respectively (Edwards *et al.*, 2017 ▸). The energies for the identified inter­molecular inter­actions are tabulated in Table 4 ▸. As anti­cipated, the greatest stabilization energy, with approximately equal contributions from *E*_ele_ and *E*_dis_, arises from the conventional amide-N—H4*N*⋯O2(carbon­yl) hydrogen bond. The next most significant energies of stabilization arise from the methyl­ene-C3—H⋯N2(azo) (dominated by *E*_dis_) and benzene-C15—H15⋯O1(amide) (approximately equal contributions from *E*_ele_ and *E*_dis_) inter­actions. In terms of energy, the next most significant contributions comes from an inter­action in the inter-layer region, namely the H17*A*⋯C12 contact, Table 4 ▸. As for the other identified inter-layer contacts, *E*_dis_ is the dominant contributor. Views of the energy framework diagrams down *a* axis are shown in Fig. 7 ▸ and confirm the crystal to be mainly stabilized by electrostatic and dispersive forces with a clear dominance from the latter. The total *E*_ele_ of all pairwise inter­actions sum to −142.9 kJ mol^−1^, while the total *E*_dis_ computes to −251.1 kJ mol^−1^.

## Database survey   

**Figure 8:**
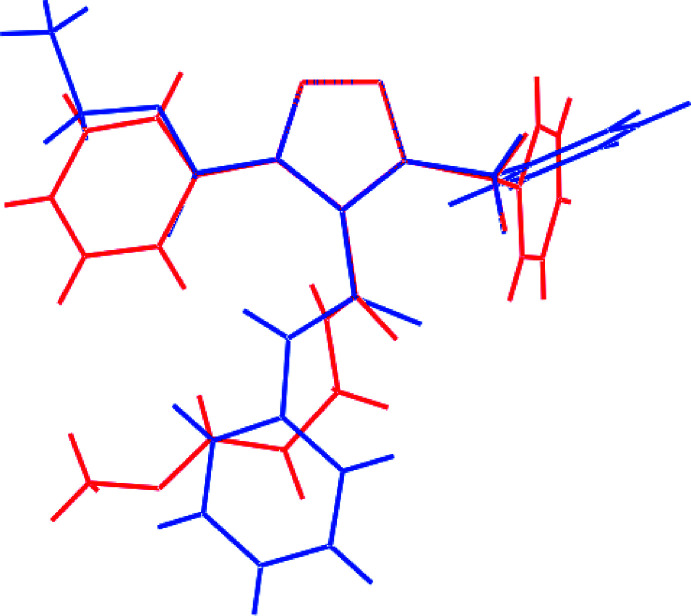
Overlay diagram for (I), red image, and (II), blue image. The mol­ecules have been overlapped so the five-membered rings are superimposed.

There is a sole literature precedent for (I), namely the analogue with ethyl carboxyl­ate and *N*-phenyl­amide substituents at the C1- and C2-atoms, respectively (WAGROM; Katritzky *et al.*, 2003 ▸), hereafter (II). An overlay diagram of (I) and (II) is given in Fig. 8 ▸. As anti­cipated, the five-membered rings and the α-atoms of the three substituents exhibit close concordance but, beyond this, the mol­ecular conformations of the terminal residues differ significantly.

## Synthesis and crystallization   

Compound (I) was prepared as described in the literature (de Albuquerque *et al.*, 2019 ▸). The crystals were obtained by the slow evaporation from an ethanol solution of (I).

## Refinement details   

Crystal data, data collection and structure refinement details are summarized in Table 5 ▸. The carbon-bound H atoms were placed in calculated positions (C—H = 0.93–0.97 Å) and were included in the refinement in the riding-model approximation, with *U*_iso_(H) set to 1.2–1.5*U*_eq_(C). The nitro­gen-bound H atom was located in a difference Fourier map and refined with N—H = 0.86±0.01 Å, and with *U*_iso_(H) set to 1.2*U*_eq_(N).

## Supplementary Material

Crystal structure: contains datablock(s) I, global. DOI: 10.1107/S2056989020007380/hb7921sup1.cif

Structure factors: contains datablock(s) I. DOI: 10.1107/S2056989020007380/hb7921Isup2.hkl

Supporting information file. DOI: 10.1107/S2056989020007380/hb7921Isup3.cml

CCDC reference: 2007664

Additional supporting information:  crystallographic information; 3D view; checkCIF report

## Figures and Tables

**Table 1:** Hydrogen-bond geometry (Å, °) *Cg*1 is the centroid of the (C10–C15) ring.

*D*—H⋯*A*	*D*—H	H⋯*A*	*D*⋯*A*	*D*—H⋯*A*
N4—H4*N*⋯O2^i^	0.86 (3)	2.04 (3)	2.884 (4)	167 (3)
C3—H3*B*⋯N2^ii^	0.97	2.55	3.495 (5)	165
C15—H15⋯O1^iii^	0.93	2.51	3.335 (5)	148
C17—H17*B*⋯*Cg*1^i^	0.97	2.71	3.640 (4)	161

Symmetry codes: (i) 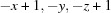; (ii) 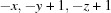; (iii) 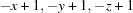.

**Table 2:** Summary of short inter­atomic contacts (Å) in (I)^*a*^

Contact	Distance	Symmetry operation
H4*N*⋯O2^*b*^	1.90	−*x* + 1, −*y*, −*z* + 1
H3*B*⋯N2^*b*^	2.44	−*x* + 1, −*y* + 1, −*z* + 1
H15⋯O1^*b*^	2.38	−*x*, −*y* + 1, −*z* + 1
H7⋯C5	2.63	−*x*, *y* − 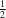 , −*z* + 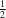
H17*A*⋯C12	2.72	*x*, −*y* + 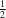 , *z* − 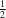
H12⋯C15	2.73	−*x* + 1, *y* − 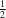 , −*z* + 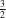

Notes: (*a*) The inter­atomic distances are calculated in *Crystal Explorer 17* (Turner *et al.*, 2017 ▸) whereby the *X*—H bond lengths are adjusted to their neutron values. (*b*) These inter­actions correspond to those reported in Table 1 ▸.

**Table 3:** Percentage contributions of inter­atomic contacts to the Hirshfeld surface for (I)

Contact	Percentage contribution
H⋯H	46.7
H⋯C/C⋯H	24.9
H⋯O/O⋯H	14.4
H⋯N/N⋯H	12.6
O⋯C/C⋯O	1.0
O⋯O	0.4

**Table 4:** Summary of inter­action energies (kJ mol^−1^) calculated for (I)

Contact	*R* (Å)	*E*_ele_	*E*_pol_	*E*_dis_	*E*_rep_	*E*_tot_
Intra-later						
N4—H4*N*⋯O2^i^	5.21	−73.7	−14.9	−84.6	94.6	−104.2
C15—H15⋯O1^iii^	5.57	−21.8	−5.7	−56.7	34.0	−55.0
C3—H3*B*⋯N2^ii^	9.97	−21.4	−6.2	−20.5	25.1	−29.6
H20*B*⋯N3^iv^ +						
H9⋯O3^v^ +						
H8⋯H18*A* ^v^	11.43	−5.1	−1.6	−14.8	6.9	−15.2
H8⋯H14^vi^	12.81	−1.4	−0.5	−9.8	2.2	−9.0
H8⋯H11^vii^	9.77	−1.2	−0.4	−8.6	1.6	−8.0
H20*C*⋯H20*C* ^viii^	14.84	1.3	−0.2	−3.1	0.6	−1.1
Inter-layer						
H17*A*⋯C12^ix^	9.37	−11.3	−3.4	−22.7	14.8	−25.1
H12⋯C15^*x*^	10.15	−5.1	−1.2	−16.3	10.0	−14.3
H7⋯C5^xi^	12.03	−3.2	−0.4	−14.0	11.5	−8.7

Notes: Symmetry operations: (i) −*x* + 1, −*y*, −*z* + 1; (ii) −*x* + 1, −*y* + 1, −*z* + 1; (iii) −*x*, −*y* + 1, −*z* + 1; (iv) *x* + 1, *y*, *z*; (v) *x* − 1, *y*, *z*; (vi) *x* − 1, −*y* + 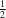, *z* − 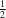; (vii) −*x*, −*y*, −*z* + 1; (viii) −*x* + 2, −*y*, −*z* + 1; (ix) *x*, −*y* + 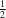, −*z* − 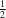; (*x*) −*x* + 1, *y* − 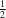, −*z* + 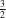; (xi) −*x*, *y* − 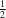, −*z* + 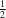.

**Table 5:** Experimental details

Crystal data	
Chemical formula	C_20_H_20_N_4_O_3_
*M*_r_	364.40
Crystal system, space group	Monoclinic, *P*2_1_/*c*
Temperature (K)	293
*a*, *b*, *c* (Å)	11.4312 (14), 9.3013 (10), 18.737 (3)
β (°)	104.695 (4)
*V* (Å^3^)	1927.1 (4)
*Z*	4
Radiation type	Mo *K*α
μ (mm^−1^)	0.09
Crystal size (mm)	0.46 × 0.31 × 0.24

Data collection	
Diffractometer	Bruker APEXII CCD
Absorption correction	Multi-scan (*SADABS*; Bruker 2009 ▸)
*T*_min_, *T*_max_	0.544, 0.745
No. of measured, independent and observed [*I* > 2σ(*I*)] reflections	24191, 3975, 2965
*R*_int_	0.066
(sin θ/λ)_max_ (Å^−1^)	0.628

Refinement	
*R*[*F* ^2^ > 2σ(*F* ^2^)], *wR*(*F* ^2^), *S*	0.077, 0.204, 1.08
No. of reflections	3975
No. of parameters	249
No. of restraints	1
H-atom treatment	H atoms treated by a mixture of independent and constrained refinement
Δρ_max_, Δρ_min_ (e Å^−3^)	0.23, −0.20

Computer programs: *APEX2* and *SAINT* (Bruker, 2009 ▸), *SIR2014* (Burla *et al.*, 2015 ▸), *SHELXL2018/3* (Sheldrick, 2015 ▸), *ORTEP-3 for Windows* (Farrugia, 2012 ▸), *DIAMOND* (Brandenburg, 2006 ▸), *MarvinSketch* (ChemAxon, 2010 ▸) and *publCIF* (Westrip, 2010 ▸).

## supplementary crystallographic information

### Crystal data

**Table:**

C_20_H_20_N_4_O_3_	*F*(000) = 768
*M**_r_* = 364.40	*D*_x_ = 1.256 Mg m^−^^3^
Monoclinic, *P*2_1_/*c*	Mo *K*α radiation, λ = 0.71073 Å
*a* = 11.4312 (14) Å	Cell parameters from 6479 reflections
*b* = 9.3013 (10) Å	θ = 2.5–23.9°
*c* = 18.737 (3) Å	µ = 0.09 mm^−^^1^
β = 104.695 (4)°	*T* = 293 K
*V* = 1927.1 (4) Å^3^	Irregular, colourless
*Z* = 4	0.46 × 0.31 × 0.24 mm

### Data collection

**Table:**

Bruker APEXII CCD diffractometer	2965 reflections with *I* > 2σ(*I*)
φ and ω scans	*R*_int_ = 0.066
Absorption correction: multi-scan (SADABS; Bruker 2009)	θ_max_ = 26.5°, θ_min_ = 1.8°
*T*_min_ = 0.544, *T*_max_ = 0.745	*h* = −14→14
24191 measured reflections	*k* = −11→11
3975 independent reflections	*l* = −23→23

### Refinement

**Table:**

Refinement on *F*^2^	Secondary atom site location: difference Fourier map
Least-squares matrix: full	Hydrogen site location: mixed
*R*[*F*^2^ > 2σ(*F*^2^)] = 0.077	H atoms treated by a mixture of independent and constrained refinement
*wR*(*F*^2^) = 0.204	*w* = 1/[σ^2^(*F*_o_^2^) + (0.0396*P*)^2^ + 3.4742*P*] where *P* = (*F*_o_^2^ + 2*F*_c_^2^)/3
*S* = 1.08	(Δ/σ)_max_ < 0.001
3975 reflections	Δρ_max_ = 0.23 e Å^−^^3^
249 parameters	Δρ_min_ = −0.20 e Å^−^^3^
1 restraint	Extinction correction: SHELXL-2018/3 (Sheldrick 2015), Fc^*^=kFc[1+0.001xFc^2^λ^3^/sin(2θ)]^-1/4^
Primary atom site location: structure-invariant direct methods	Extinction coefficient: 0.0267 (19)

### Special details

**Table:**

Geometry. All esds (except the esd in the dihedral angle between two l.s. planes) are estimated using the full covariance matrix. The cell esds are taken into account individually in the estimation of esds in distances, angles and torsion angles; correlations between esds in cell parameters are only used when they are defined by crystal symmetry. An approximate (isotropic) treatment of cell esds is used for estimating esds involving l.s. planes.

### Fractional atomic coordinates and isotropic or equivalent isotropic displacement parameters (Å^2^)

**Table:**

	*x*	*y*	*z*	*U*_iso_*/*U*_eq_	
O1	0.3969 (2)	0.4513 (3)	0.43041 (14)	0.0558 (7)	
O2	0.6787 (3)	0.0379 (3)	0.5049 (2)	0.0836 (10)	
O3	0.8198 (3)	0.2035 (3)	0.5160 (2)	0.0829 (10)	
N1	0.1976 (2)	0.4009 (3)	0.49963 (14)	0.0450 (7)	
N2	0.1426 (3)	0.3888 (4)	0.55460 (17)	0.0635 (9)	
N3	0.2173 (3)	0.3227 (4)	0.60901 (16)	0.0598 (9)	
N4	0.4356 (2)	0.2164 (3)	0.45617 (14)	0.0409 (6)	
H4N	0.409 (3)	0.143 (2)	0.4749 (18)	0.049*	
C1	0.3218 (3)	0.2907 (3)	0.58938 (16)	0.0399 (7)	
C2	0.3086 (3)	0.3412 (3)	0.51805 (16)	0.0370 (7)	
C3	0.1285 (3)	0.4596 (4)	0.42786 (19)	0.0527 (9)	
H3A	0.180906	0.520492	0.407502	0.063*	
H3B	0.062468	0.518335	0.435328	0.063*	
C4	0.0785 (3)	0.3410 (4)	0.37406 (18)	0.0482 (8)	
C5	0.1396 (4)	0.2962 (5)	0.3228 (2)	0.0691 (12)	
H5	0.212880	0.338684	0.321872	0.083*	
C6	0.0922 (6)	0.1895 (7)	0.2736 (2)	0.0986 (19)	
H6	0.133334	0.158992	0.239445	0.118*	
C7	−0.0168 (7)	0.1276 (7)	0.2749 (3)	0.111 (2)	
H7	−0.049067	0.054764	0.241663	0.133*	
C8	−0.0772 (6)	0.1728 (6)	0.3245 (3)	0.1071 (19)	
H8	−0.151197	0.131428	0.324646	0.128*	
C9	−0.0299 (4)	0.2792 (5)	0.3747 (2)	0.0707 (12)	
H9	−0.071464	0.308948	0.408779	0.085*	
C10	0.4213 (3)	0.2167 (3)	0.64097 (16)	0.0410 (7)	
C11	0.3958 (4)	0.1262 (4)	0.69416 (19)	0.0598 (10)	
H11	0.316046	0.112350	0.695971	0.072*	
C12	0.4876 (6)	0.0571 (5)	0.7442 (2)	0.0812 (15)	
H12	0.469542	−0.003111	0.779513	0.097*	
C13	0.6056 (5)	0.0765 (5)	0.7421 (2)	0.0787 (15)	
H13	0.667277	0.028938	0.775749	0.094*	
C14	0.6325 (4)	0.1658 (5)	0.6906 (2)	0.0679 (12)	
H14	0.712690	0.179290	0.689602	0.081*	
C15	0.5411 (3)	0.2367 (4)	0.63968 (18)	0.0489 (8)	
H15	0.560139	0.297321	0.604805	0.059*	
C16	0.3854 (3)	0.3416 (3)	0.46468 (16)	0.0357 (7)	
C17	0.5073 (3)	0.1985 (4)	0.40222 (19)	0.0560 (10)	
H17A	0.469697	0.251931	0.357879	0.067*	
H17B	0.508059	0.097734	0.389017	0.067*	
C18	0.6360 (3)	0.2500 (4)	0.4315 (2)	0.0601 (10)	
H18A	0.673282	0.260002	0.390743	0.072*	
H18B	0.634970	0.344092	0.453692	0.072*	
C19	0.7104 (3)	0.1507 (4)	0.4873 (2)	0.0525 (9)	
C20	0.9011 (5)	0.1147 (7)	0.5700 (4)	0.116 (2)	
H20A	0.864583	0.092823	0.609558	0.173*	
H20B	0.975609	0.165410	0.589204	0.173*	
H20C	0.917047	0.027061	0.547137	0.173*	

### Atomic displacement parameters (Å^2^)

**Table:**

	*U*^11^	*U*^22^	*U*^33^	*U*^12^	*U*^13^	*U*^23^
O1	0.0565 (15)	0.0461 (14)	0.0659 (16)	−0.0003 (11)	0.0177 (12)	0.0213 (12)
O2	0.079 (2)	0.0530 (17)	0.114 (3)	−0.0147 (15)	0.0151 (18)	0.0224 (17)
O3	0.0569 (17)	0.071 (2)	0.116 (3)	−0.0102 (15)	0.0128 (17)	0.0251 (18)
N1	0.0430 (15)	0.0491 (16)	0.0420 (14)	0.0096 (13)	0.0092 (12)	−0.0051 (12)
N2	0.0502 (18)	0.092 (3)	0.0525 (18)	0.0146 (18)	0.0204 (15)	−0.0114 (18)
N3	0.0558 (18)	0.082 (2)	0.0465 (17)	0.0083 (17)	0.0218 (14)	−0.0051 (16)
N4	0.0478 (15)	0.0374 (14)	0.0422 (14)	−0.0016 (12)	0.0203 (12)	0.0002 (12)
C1	0.0442 (17)	0.0411 (17)	0.0365 (15)	0.0006 (14)	0.0140 (13)	−0.0057 (13)
C2	0.0396 (16)	0.0311 (15)	0.0399 (16)	0.0042 (13)	0.0094 (12)	−0.0019 (13)
C3	0.050 (2)	0.048 (2)	0.055 (2)	0.0155 (16)	0.0042 (16)	0.0014 (16)
C4	0.0507 (19)	0.0495 (19)	0.0407 (17)	0.0144 (16)	0.0048 (14)	0.0016 (15)
C5	0.076 (3)	0.084 (3)	0.049 (2)	0.021 (2)	0.018 (2)	0.001 (2)
C6	0.128 (5)	0.113 (5)	0.048 (2)	0.037 (4)	0.009 (3)	−0.024 (3)
C7	0.133 (5)	0.096 (4)	0.079 (4)	0.006 (4)	−0.019 (4)	−0.041 (3)
C8	0.108 (4)	0.094 (4)	0.104 (4)	−0.032 (3)	−0.001 (4)	−0.024 (4)
C9	0.062 (3)	0.082 (3)	0.064 (2)	−0.007 (2)	0.009 (2)	−0.014 (2)
C10	0.059 (2)	0.0350 (16)	0.0297 (14)	0.0017 (14)	0.0129 (13)	−0.0044 (12)
C11	0.093 (3)	0.052 (2)	0.0417 (19)	0.000 (2)	0.0307 (19)	0.0006 (16)
C12	0.148 (5)	0.060 (3)	0.042 (2)	0.021 (3)	0.035 (3)	0.0155 (19)
C13	0.119 (4)	0.070 (3)	0.038 (2)	0.037 (3)	0.002 (2)	0.0025 (19)
C14	0.064 (2)	0.078 (3)	0.052 (2)	0.019 (2)	−0.0025 (18)	−0.005 (2)
C15	0.057 (2)	0.048 (2)	0.0388 (17)	0.0015 (16)	0.0058 (15)	0.0003 (15)
C16	0.0341 (15)	0.0349 (16)	0.0360 (15)	−0.0021 (13)	0.0053 (12)	0.0029 (13)
C17	0.059 (2)	0.069 (2)	0.0475 (19)	0.0052 (19)	0.0267 (17)	−0.0019 (17)
C18	0.058 (2)	0.059 (2)	0.072 (2)	0.0023 (19)	0.034 (2)	0.015 (2)
C19	0.055 (2)	0.044 (2)	0.066 (2)	−0.0056 (17)	0.0293 (18)	0.0014 (17)
C20	0.068 (3)	0.119 (5)	0.145 (5)	0.011 (3)	0.000 (3)	0.036 (4)

### Geometric parameters (Å, º)

**Table:**

O1—C16	1.231 (4)	C7—H7	0.9300
O2—C19	1.184 (4)	C8—C9	1.378 (7)
O3—C19	1.324 (5)	C8—H8	0.9300
O3—C20	1.446 (6)	C9—H9	0.9300
N1—N2	1.341 (4)	C10—C15	1.388 (5)
N1—C2	1.347 (4)	C10—C11	1.391 (5)
N1—C3	1.480 (4)	C11—C12	1.376 (6)
N2—N3	1.306 (4)	C11—H11	0.9300
N3—C1	1.368 (4)	C12—C13	1.371 (7)
N4—C16	1.326 (4)	C12—H12	0.9300
N4—C17	1.464 (4)	C13—C14	1.366 (6)
N4—H4N	0.860 (10)	C13—H13	0.9300
C1—C2	1.388 (4)	C14—C15	1.389 (5)
C1—C10	1.464 (4)	C14—H14	0.9300
C2—C16	1.489 (4)	C15—H15	0.9300
C3—C4	1.506 (5)	C17—C18	1.511 (5)
C3—H3A	0.9700	C17—H17A	0.9700
C3—H3B	0.9700	C17—H17B	0.9700
C4—C9	1.368 (5)	C18—C19	1.489 (5)
C4—C5	1.387 (5)	C18—H18A	0.9700
C5—C6	1.370 (7)	C18—H18B	0.9700
C5—H5	0.9300	C20—H20A	0.9600
C6—C7	1.378 (8)	C20—H20B	0.9600
C6—H6	0.9300	C20—H20C	0.9600
C7—C8	1.358 (8)		
			
C19—O3—C20	116.5 (4)	C11—C10—C1	119.2 (3)
N2—N1—C2	111.3 (3)	C12—C11—C10	120.5 (4)
N2—N1—C3	118.9 (3)	C12—C11—H11	119.7
C2—N1—C3	129.5 (3)	C10—C11—H11	119.7
N3—N2—N1	107.3 (3)	C13—C12—C11	120.3 (4)
N2—N3—C1	109.6 (3)	C13—C12—H12	119.8
C16—N4—C17	121.3 (3)	C11—C12—H12	119.8
C16—N4—H4N	116 (2)	C14—C13—C12	120.0 (4)
C17—N4—H4N	121 (2)	C14—C13—H13	120.0
N3—C1—C2	107.2 (3)	C12—C13—H13	120.0
N3—C1—C10	120.6 (3)	C13—C14—C15	120.6 (4)
C2—C1—C10	132.1 (3)	C13—C14—H14	119.7
N1—C2—C1	104.6 (3)	C15—C14—H14	119.7
N1—C2—C16	120.3 (3)	C10—C15—C14	119.9 (4)
C1—C2—C16	135.2 (3)	C10—C15—H15	120.1
N1—C3—C4	111.3 (3)	C14—C15—H15	120.1
N1—C3—H3A	109.4	O1—C16—N4	123.9 (3)
C4—C3—H3A	109.4	O1—C16—C2	120.8 (3)
N1—C3—H3B	109.4	N4—C16—C2	115.2 (3)
C4—C3—H3B	109.4	N4—C17—C18	112.1 (3)
H3A—C3—H3B	108.0	N4—C17—H17A	109.2
C9—C4—C5	119.7 (4)	C18—C17—H17A	109.2
C9—C4—C3	119.5 (3)	N4—C17—H17B	109.2
C5—C4—C3	120.7 (4)	C18—C17—H17B	109.2
C6—C5—C4	120.0 (5)	H17A—C17—H17B	107.9
C6—C5—H5	120.0	C19—C18—C17	112.9 (3)
C4—C5—H5	120.0	C19—C18—H18A	109.0
C5—C6—C7	119.8 (5)	C17—C18—H18A	109.0
C5—C6—H6	120.1	C19—C18—H18B	109.0
C7—C6—H6	120.1	C17—C18—H18B	109.0
C8—C7—C6	120.0 (5)	H18A—C18—H18B	107.8
C8—C7—H7	120.0	O2—C19—O3	122.7 (4)
C6—C7—H7	120.0	O2—C19—C18	125.7 (4)
C7—C8—C9	120.7 (6)	O3—C19—C18	111.6 (3)
C7—C8—H8	119.7	O3—C20—H20A	109.5
C9—C8—H8	119.7	O3—C20—H20B	109.5
C4—C9—C8	119.7 (5)	H20A—C20—H20B	109.5
C4—C9—H9	120.1	O3—C20—H20C	109.5
C8—C9—H9	120.1	H20A—C20—H20C	109.5
C15—C10—C11	118.7 (3)	H20B—C20—H20C	109.5
C15—C10—C1	122.0 (3)		
			
C2—N1—N2—N3	0.5 (4)	N3—C1—C10—C15	−150.9 (3)
C3—N1—N2—N3	174.1 (3)	C2—C1—C10—C15	28.8 (5)
N1—N2—N3—C1	−0.3 (4)	N3—C1—C10—C11	27.5 (5)
N2—N3—C1—C2	−0.1 (4)	C2—C1—C10—C11	−152.8 (4)
N2—N3—C1—C10	179.6 (3)	C15—C10—C11—C12	−0.5 (5)
N2—N1—C2—C1	−0.6 (4)	C1—C10—C11—C12	−178.9 (3)
C3—N1—C2—C1	−173.3 (3)	C10—C11—C12—C13	0.0 (6)
N2—N1—C2—C16	179.6 (3)	C11—C12—C13—C14	0.5 (7)
C3—N1—C2—C16	6.8 (5)	C12—C13—C14—C15	−0.5 (6)
N3—C1—C2—N1	0.4 (4)	C11—C10—C15—C14	0.5 (5)
C10—C1—C2—N1	−179.3 (3)	C1—C10—C15—C14	178.9 (3)
N3—C1—C2—C16	−179.8 (3)	C13—C14—C15—C10	0.0 (6)
C10—C1—C2—C16	0.6 (6)	C17—N4—C16—O1	−2.0 (5)
N2—N1—C3—C4	−97.4 (4)	C17—N4—C16—C2	176.2 (3)
C2—N1—C3—C4	74.8 (5)	N1—C2—C16—O1	46.0 (4)
N1—C3—C4—C9	85.4 (4)	C1—C2—C16—O1	−133.9 (4)
N1—C3—C4—C5	−96.2 (4)	N1—C2—C16—N4	−132.3 (3)
C9—C4—C5—C6	−0.7 (6)	C1—C2—C16—N4	47.9 (5)
C3—C4—C5—C6	−179.1 (4)	C16—N4—C17—C18	82.3 (4)
C4—C5—C6—C7	0.4 (8)	N4—C17—C18—C19	73.6 (4)
C5—C6—C7—C8	0.3 (9)	C20—O3—C19—O2	0.8 (7)
C6—C7—C8—C9	−0.8 (10)	C20—O3—C19—C18	−178.9 (4)
C5—C4—C9—C8	0.2 (7)	C17—C18—C19—O2	5.0 (6)
C3—C4—C9—C8	178.6 (4)	C17—C18—C19—O3	−175.4 (3)
C7—C8—C9—C4	0.6 (9)		

### Hydrogen-bond geometry (Å, º)

*Cg*1 is the centroid of the (C10–C15) ring.

**Table:**

*D*—H···*A*	*D*—H	H···*A*	*D*···*A*	*D*—H···*A*
N4—H4*N*···O2^i^	0.86 (3)	2.04 (3)	2.884 (4)	167 (3)
C3—H3*B*···N2^ii^	0.97	2.55	3.495 (5)	165
C15—H15···O1^iii^	0.93	2.51	3.335 (5)	148
C17—H17*B*···*Cg*1^i^	0.97	2.71	3.640 (4)	161

Symmetry codes: (i) −*x*+1, −*y*, −*z*+1; (ii) −*x*, −*y*+1, −*z*+1; (iii) −*x*+1, −*y*+1, −*z*+1.
